# Analysis of Hypericin-Mediated Effects and Implications for Targeted Photodynamic Therapy

**DOI:** 10.3390/ijms18071388

**Published:** 2017-06-29

**Authors:** Laura Mühleisen, Magdalena Alev, Harald Unterweger, Daniel Subatzus, Marina Pöttler, Ralf P. Friedrich, Christoph Alexiou, Christina Janko

**Affiliations:** 1 Department of Otorhinolaryngology, Head and Neck Surgery, Section of Experimental Oncology and Nanomedicine (SEON), Else Kröner-Fresenius-Stiftung-Professorship, Universitätsklinikum Erlangen, 91054 Erlangen, Germany; laura.muehleisen@web.de (L.M.); magdalena_alev@hotmail.com (M.A.); harald.unterweger@uk-erlangen.de (H.U.); dani_subatzus@yahoo.de (D.S.); marina.poettler@uk-erlangen.de (M.P.); ralf.friedrich@uk-erlangen.de (R.P.F.); christoph.alexiou@uk-erlangen.de (C.A.); 2 Friedrich-Alexander-Universität Erlangen-Nürnberg (FAU), 91054 Erlangen, Germany

**Keywords:** nanomedicine, hypericin, magnetic drug targeting (MDT), photodynamic therapy (PDT), superparamagnetic iron oxide nanoparticles (SPION)

## Abstract

The phototoxic effect of hypericin can be utilized for Photodynamic Therapy (PDT) of cancer. After intravenous application and systemic distribution of the drug in the patient’s body, the tumor site is exposed to light. Subsequently, toxic reactive oxygen species (ROS) are generated, inducing tumor cell death. To prevent unwanted activation of the drug in other regions of the body, patients have to avoid light during and after the treatment cycles, consequently impairing quality of life. Here, we characterize toxicity and hypericin-mediated effects on cancer cells in vitro and confirm that its effect clearly depends on concentration and illumination time. To reduce side effects and to increase therapy success, selective accumulation of hypericin in the tumor region is a promising solution. Loading hypericin on superparamagnetic iron oxide nanoparticles (SPIONs) and guiding them to the desired place using an external magnetic field might accomplish this task (referred to as Magnetic Drug Targeting (MDT)). Thus, using a double targeting strategy, namely magnetic accumulation and laser induced photoactivation, might improve treatment effectivity as well as specificity and reduce toxic side effects in future clinical applications.

## 1. Introduction

Chemotherapy in cancer treatment is often accompanied by side effects, since intravenously applied cytotoxic drugs do not only accumulate in tumor tissues but distribute within the whole body [1]. Adverse reactions of common chemotherapeutic agents, for instance, comprise vomiting, nausea, cardiotoxicity, and immune suppression [1,2,3]. To restrict cytotoxic effects to the tumor region, a more targeted approach would be desirable. Thus, the targeting of active toxic substances to the tumor region or activation of initially inactive substances in the tumor region might be a solution to overcome this problem.

Hypericin, naturally occurring as a component of St. John’s wort (*Hypericum perforatum*) and often used as anti-depressive agent, reveals photoactive features, with low toxicity in the absence of light [4,5]. In the presence of light (absorption peaks at 545 and 590 nm), however, toxic singlet oxygen and radical species are produced due to its extended π-electron system [6,7,8]. Thus, hypericin has been used as a photosensitizer in the photodynamic therapy (PDT) of dermatosis as well as for superficial cancers like mucosa and skin cancer [9]. For therapy, the patient systemically receives a photosensitizing drug, and external lesions are subsequently irradiated by laser light of suitable wavelengths, whereas internal cancer types must be illuminated via endoscopy [10,11,12]. Although PDT is less invasive than conventional chemotherapy, there are still side effects that limit the patients’ quality of life. Due to the systemic distribution of the photosensitizer after intravenous application, patients suffer from photosensitivity, a pathological light hypersensitivity of the skin and retina, which forces patients after PDT to avoid daylight for weeks, otherwise symptoms like sunburn, redness, and sharp pain can occur [13,14]. Also, some light independent effects have been described previously [5,15]. Hypericin is taken up by tumor cells via endocytosis, pinocytosis, or passive diffusion. Intracellularly it accumulates in the endoplasmic reticulum and Golgi apparatus, where reactive oxygen species (ROS) cause local cell damage, and, depending on available light dose and concentration of hypericin, this leads to apoptosis or necrosis of tumor cells [4,9,14,16,17,18,19,20].

**Figure 1:**
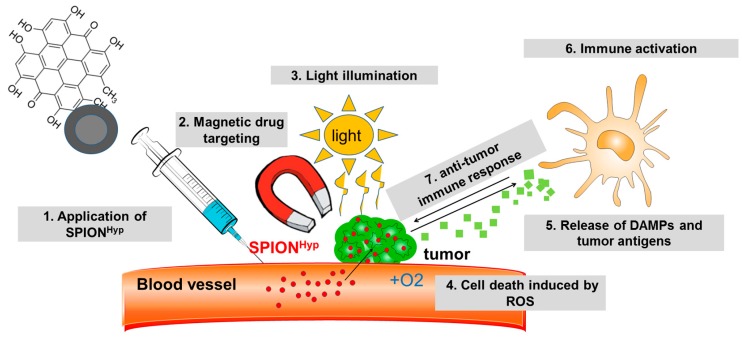
A combination of Magnetic Drug Targeting (MDT) and Photodynamic Therapy (PDT). Superparamagnetic iron oxide nanoparticles loaded with hypericin (SPION^Hyp^) are applied intra-arterially into the tumor-supplying vascular system (**1**) and enriched in the tumor region using an external magnetic field (**2**). With light illumination (**3**), hypericin is activated and induces cell death by reactive oxygen species (ROS) (**4**). Cell death is accompanied by the release of antigens and damage associated molecular patterns (DAMPs) (**5**), fostering antigen-presenting cells to take up tumor antigens and to cross-present them to T cells (**6**). Finally, a long-term anti-tumor response by the immune system is induced (**7**).

To overcome unspecific distribution of therapeutics, we previously developed superparamagnetic iron oxide nanoparticles (SPIONs) for targeted delivery of drugs to the tumor region by means of an external magnetic field referred to as “Magnetic Drug Targeting” (MDT) [21]. Promising therapeutic outcomes have been shown, for instance, for mitoxantrone-loaded nanoparticles in tumor bearing rabbits by us previously [22], and also other groups have successfully targeted tumors with SPIONs using magnetic forces [23,24]. Loading hypericin onto SPIONs (SPION^Hyp^) and guiding them along to the desired place using an external magnetic field is supposed to increase tumor targeting and lower phototoxic side effects [25]. Figure 1 depicts how a combination of MDT and PDT is intended to work. SPION^Hyp^ are applied intra-arterially into the tumor supplying vascular system and enriched in the tumor region by an external magnetic field. When illuminated, intratumoral hypericin is activated and induces cell death concomitant to ROS production. Hypericin is known to induce an immunogenic cell death phenotype which is accompanied by the release of damage associated molecular patterns (DAMPs), acting as endogenous adjuvants [26,27]. Subsequently, antigen-presenting cells are stimulated to take up tumor antigens, to cross-present them, and to activate T cells to mediate a long-term anti-tumor response.

In this work, we analyze the efficacy of hypericin on tumor cell killing and provide insights into the induced cell death phenotype. We also provide an outlook in the targeted delivery of hypericin in cancer treatment using nanotechnology.

## 2. Results

### 2.1. Intracellular Accumulation of Hypericin

**Figure 2:**
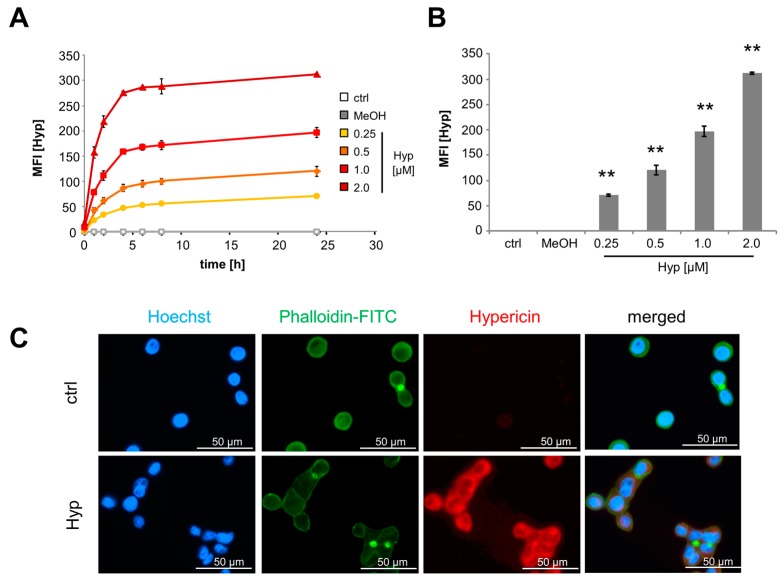
Cellular hypericin uptake is dose and time dependent. (**A**) Kinetics of hypericin uptake in Jurkat cells measured by flow cytometry and (**B**) summarized after 24 h. Shown are the mean values ± standard deviations of quadruplicates of one representative experiment (Student’s *t*-test * *p* < 0.05; ** *p* < 0.01, treated cells versus control cells). (**C**) Fluorescence microscopy of HT-29 cells after incubation for 24 h with 2 μM hypericin (**red**); cellular nuclei and cytoskeleton were stained with Hoechst33342 (**blue**) and phalloidin-FITC (**green**). The scale bars represent 50 µm.

Hypericin can freely diffuse into cells or be incorporated by endocytosis [19,20], and due to its inherent fluorescence intracellular hypericin can easily be monitored by fluorescence microscopy or flow cytometry. To analyze diffusion velocity of hypericin into cells, we incubated Jurkat cells with different hypericin concentrations and measured hypericin fluorescence using flow cytometry. Untreated cells as well as cells treated with methanol as solvent for hypericin served as controls. To avoid phototoxicity, cells were kept in the dark during the experiment. In all conditions hypericin fluorescence increased remarkably in the first 6 h after treatment. After that, all samples only showed a slight increase in fluorescence (Figure 2A). Dependent on the hypericin concentration added into the cell culture medium, cells revealed a significant dose-dependent increase of intracellular hypericin after 24 h incubation time (Figure 2B). This was also confirmed in HT-29 cells, which were incubated with hypericin for 24 h and subsequently analyzed in fluorescence microscopy (Figure 2C). The cytoskeleton was stained with phalloidin-FITC and the nuclei with Hoechst 33342, as shown by green and blue fluorescence, respectively. Hypericin fluorescence is depicted in red. The merged picture clearly demonstrates that hypericin primarily localizes in the cytoplasm and then most likely, due to previous reports, in the endoplasmic reticulum, lysosomes, and Golgi apparatus [19,20,28].

### 2.2. Oxidative Stress Induced by Hypericin

**Figure 3:**
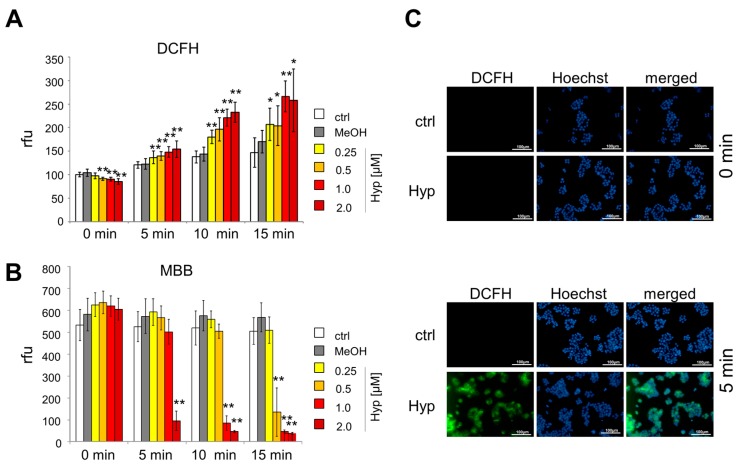
Hypericin induces oxidative stress dependent on concentration and illumination time. Jurkat cells were incubated for 12 h with hypericin and stained (**A**) for ROS using 2′,7′-dichlorodihydrofluorescein diacetate (DCFH-DA) or (**B**) for reduced glutathione using monobromobimane (MBB). Shown are the mean values ± standard deviations of *n* = 3 independent experiments with technical triplicates (Student’s *t*-test * *p* < 0.05; ** *p* < 0.01, treated cells versus control cells at the respective illumination times); (**C**) fluorescence microscopy of HT-29 cells incubated with 2 μM hypericin for 12 h after 5 min illumination. Cellular nuclei were stained with Hoechst 33342 (**blue**) and ROS was visualized with DCFH-DA (**green**). The scale bars represent 100 µm.

Previously, it has been shown that hypericin elicits low toxicity in the dark, but when illuminated with light, reactive oxygen species (ROS) are generated, which can damage cellular macromolecules as DNA, lipids, or proteins. By staining with 2′,7′-dichlorodihydrofluorescein diacetate (DCFH-DA) and monobromobimane (MBB), we analyzed cellular oxygen and nitrogen radicals as well as the redox status of glutathione [29,30]. Non-polar DCFH-DA is taken up by cells and converted into polar non-fluorescent DCFH by esterases. As soon as it is oxidized by ROS to DCF, a bright green fluorescence can be detected [31]. As an antioxidant, glutathione serves as an electron donor and is oxidized to glutathione disulfide (GSSG) in the presence of ROS. Initially non-fluorescent MBB reacts with thiols, and shows its brightest fluorescent at a high level of non-oxidized glutathione (GSH) [32,33]. To enable proper cellular penetration, Jurkat cells were incubated with hypericin for 12 h overnight and then illuminated with light for 5, 10, or 15 min. Non-illuminated cells served as controls. DCFfluorescence showed a significant increase of ROS dependent on illumination time and hypericin concentration (Figure 3A). At the same time, the MBB fluorescence decreased, thereby indicating oxidation of glutathione (Figure 3B). Data were confirmed by fluorescence microscopy using HT-29 cells which were incubated with hypericin overnight, illuminated for 5 min, and stained with DCFH-DA and Hoechst 33342 for the imaging of ROS and cellular nuclei. Using fluorescence microscopy, no green DCF fluorescence was detected in cells kept in the dark, whereas a clear green fluorescence indicated ROS in cells after illumination (Figure 3C).

### 2.3. Inhibition of Cell Proliferation and Induction of Cell Death by Hypericin

**Figure 4:**
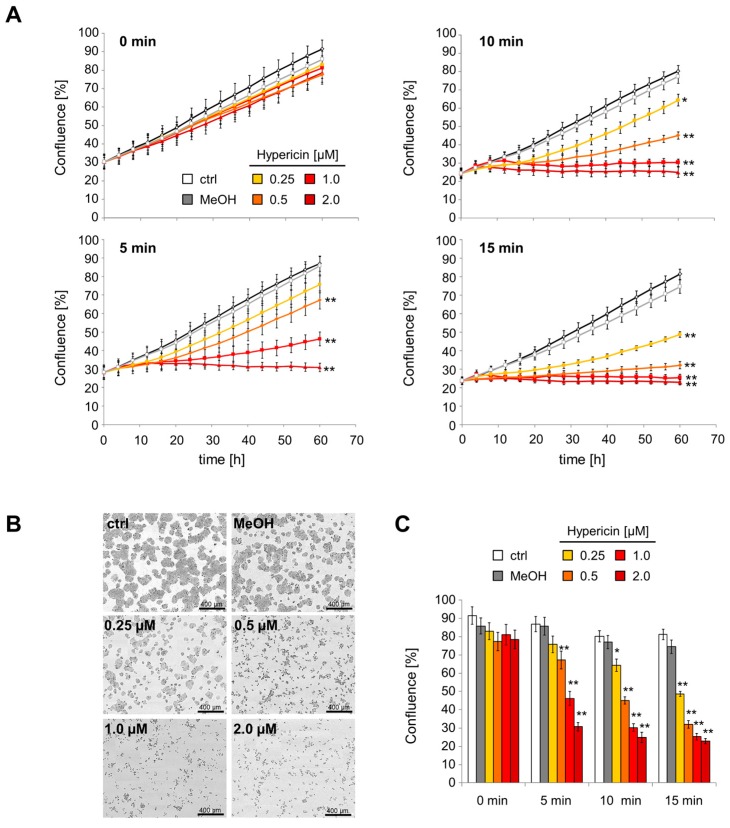
Hypericin reduces cell proliferation dependent on dose and illumination time. HT-29 cells were treated with hypericin for 12 h, illuminated, and monitored using the IncuCyte^®^ live cell analysis system. (**A**) Kinetics of cell confluences was calculated from raw data images; (**B**) raw data pictures of cell confluence exported from the IncuCyte^®^ system after 60 h incubation (15 min illumination); the scale bars represent 400 µm; (**C**) cell confluences after 60 h of incubation. Shown are the mean values ± standard deviations of *n* = 3 independent experiments with technical quadruplicates (Student’s *t*-test * *p* < 0.05; ** *p* < 0.01, treated cells versus control cells at the respective illumination times).

Using the IncuCyte^®^ live cell analysis system, we monitored the proliferation of hypericin-treated HT-29 cells over 60 h. Without illumination, the cell confluence continuously increased and only minor differences between hypericin-treated cells and controls were detected. After illumination, however, hypericin decreased proliferation rates depending on illumination times and hypericin concentrations. After treatment with 2 μM hypericin, the cell proliferation was completely abolished for all investigated illumination times (Figure 4A). After 60 h, significant differences in cell confluence were detected (Figure 4B,C). Whereas the proliferation of untreated and methanol-treated cells was not influenced by illumination, the proliferation of hypericin-treated cells was significantly reduced and even completely abolished for high hypericin concentrations and long illumination times.

**Figure 5:**
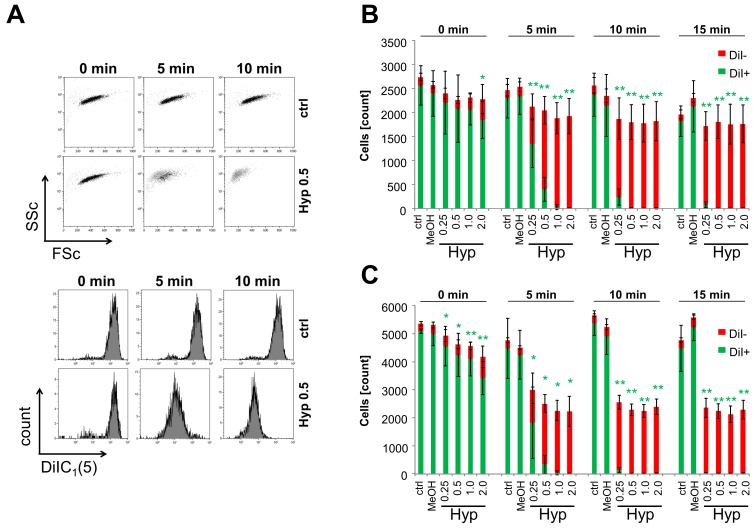
Hypericin induces cell death dependent on concentration and illumination time. Jurkat cells were incubated with hypericin for 12 h, illuminated with light, and analyzed after 8 and 24 h in flow cytometry. (**A**) Flow cytometry raw data files of Jurkat cells (*t* = 0 h after illumination; 0.5 μM Hyp). Forward (FSc) and side scatter (SSc) (upper panel) as well as staining for mitochondrial membrane potential with the hexamethylindodicarbocyanine iodide dye DiIC_1_(5) (lower panel) provide information about cellular viability. Bar graphs show cell count after 8 h (**B**) and 24 h (**C**) for DiIC_1_(5)+ (viable, **green**) and DiIC_1_(5)− (dead, **red**) cells. Shown are the mean values ± standard deviations of *n* = 4 independent experiments performed in duplicates (ctrl, H_2_O) or quadruplicates (Hyp) (Student’s *t*-test, * *p* < 0.05; ** *p* < 0.01, referring to DiIC_1_(5)+ cells, treated cells versus control cells at the respective illumination times).

To further characterize hypericin-mediated effects, Jurkat cells were stained for mitochondrial membrane potential using the hexamethylindodicarbocyanine dye DiIC_1_(5) after 8 and 24 h of hypericin treatment with and without light illumination. Figure 5A exemplarily depicts flow cytometry raw data files for 0.5 μM hypericin in the presence and absence of light. Dying and dead cells can be clearly differentiated from viable cells by their morphological features (upper panel) and staining properties (lower panel, Figure 5A). Forward (FSc) and side scatter (SSc) provide information of cellular size and granularity, which are characteristically altered during cell death (after illumination of hypericin-treated cells, upper panel). As long as a proton gradient exists at the mitochondrial membrane, DiIC_1_(5) fluorescence indicates metabolically healthy viable cells (lower panel) [34]. During the 24 h observation time the untreated cells proliferated in the presence or absence of light. With increasing hypericin concentration and illumination times the total number of viable cells decreased and with illumination ≥10 min, no viable cells were remaining. In contrast, illumination for 5 min at low hypericin concentration led to incomplete killing of cells and proliferation of remaining viable cells after 24 h (Figure 5B,C).

### 2.4. Phagocytosis of Hypericin-Treated JURKAT Cells by Macrophages

**Figure 6:**
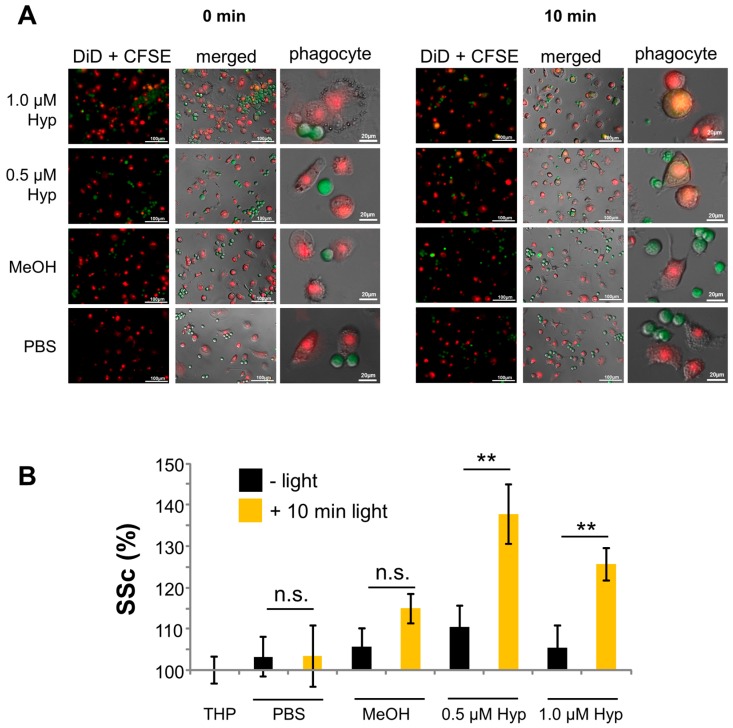
Phagocytosis of hypericin-treated Jurkat cells by macrophages. (**A**) Fluorescence microscopy of phagocytosis of hypericin-treated Jurkat cells (stained with carboxyfluorescein succinimidyl ester (CFSE, **green**) by macrophages (stained with DiD (C_67_H_103_ClN_2_O_3_S), **red**); the scale bar represents 100 µm (overview pictures left and middle) or 20 µm (magnification, right); (**B**) Flow cytometry of macrophages with ingested Jurkat cells. The side scatter (SSc) reflects cellular granularity and serves as a marker to estimate phagocytosis. Shown are the mean values of triplicates (Student’s *t*-test, * *p* < 0.05; ** *p* < 0.01, non-illuminated versus illuminated cells).

Hypericin has previously been shown to induce immunogenic cell death accompanied by exposition of “eat-me” signals and release of danger signals. This leads to fast and efficient uptake of dying and dead cells by professional phagocytes and antigen presenting cells [27,35,36]. THP monocytes (stained with DiD (C_67_H_103_ClN_2_O_3_S), depicted in red) were differentiated into macrophages for 3 days and subsequently incubated with hypericin-treated and non-illuminated or light-illuminated Jurkat cells (stained with carboxyfluorescein succinimidyl ester (CFSE), depicted in green). Fluorescence microscopy showed that Jurkat cells which had been treated with 0.5 and 1 μM hypericin (without illumination) are not taken up by macrophages, as shown by non-colocalizing green and red fluorescences. As soon as cells were illuminated, phagocytosis was increased, as shown by colocalizing green and red fluorescence (Figure 6A). To prove this result, we analyzed phagocytosis of Jurkat cells by macrophages in flow cytometry (Figure 6B). For detection of phagocytosis, side scatter of viable monocytes (DiD+ cells) was analyzed, because the side scatter provides information about cellular granularity, which increases when cells take up, for example, bacteria, nanoparticles, or dying cells [37,38,39,40]. Side scatter values of untreated macrophages were normalized to 100% and side scatter of treated ones was calculated accordingly. After illumination, the uptake of Jurkat control cells as well as methanol-treated cells was only slightly increased, whereas the uptake of hypericin-treated cells was strongly increased (Figure 6B).

### 2.5. Comparison of Free Hypericin and SPION^Hyp^

**Figure 7:**
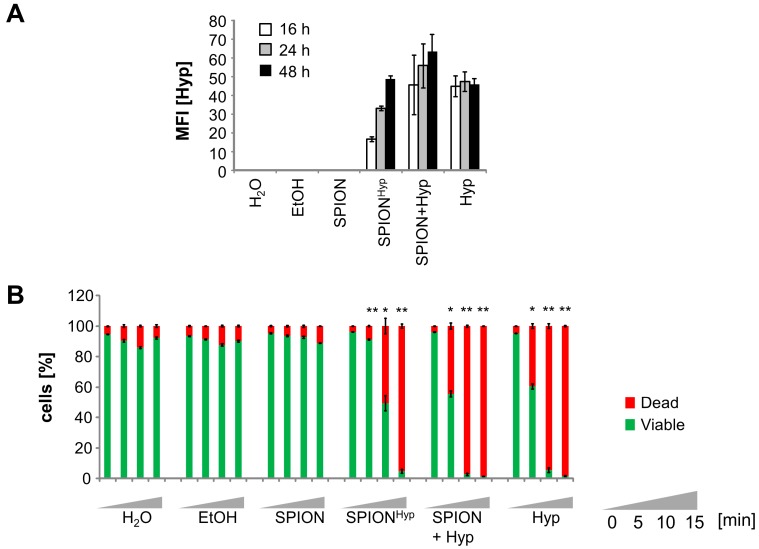
Comparison of Jurkat cells treated with SPION^Hyp^, SPION+Hyp, free Hyp: (**A**) Time dependent intracellular hypericin fluorescence (mean fluorescence index, MFI), measured at 16 h, 24 h, and 48 h without illumination (Hyp concentration = 0.4 μM); (**B**) comparison of cellular viability after different treatments and illumination times after 16 h. Shown are the mean values of triplicates (Student’s *t*-test, * *p* < 0.01; ** *p* < 0.005; non-illuminated versus illuminated cells at the respective treatment).

To increase drug accumulation in the tumor and to reduce systemic side effects in healthy tissues, hypericin was loaded onto superparamagnetic iron oxide nanoparticles (SPIONs) as previously described [25], referred to as SPION^Hyp^. Free hypericin and SPIONs in the presence of free hypericin were analyzed in parallel. Intracellular hypericin fluorescence was measured 16, 24, and 48 h after the addition of the respective substances (Figure 7A). As shown before (Figure 2), the fluorescence of intracellular free hypericin remains constant after 16 h. When cells were treated with SPION^Hyp^, there was only a weak fluorescence after 16 h, which increased up to the level of free hypericin after 48 h, probably due to ongoing endocytosis. Incubating cells with free hypericin in the presence of nanoparticles further elevated the uptake of the substance, which may be explained by additional co-ingestion of the free drug when nanoparticles are taken up by endocytosis (Figure 7A).

Sixteen hours after illumination for 0, 5, 10, or 15 min a clear light-dependent effect on cell viability was determined in flow cytometry. Without illumination, free hypericin and SPION^Hyp^ did not show any toxicity. As soon as cells were illuminated, cell death was induced. Here, only a 15-min illumination was sufficient to reliably kill all SPION^Hyp^ treated cells, whereas for the other hypericin conditions, 10 min of illumination was sufficient. This finding reflects the different kinetics and efficacies in cellular uptake of hypericin loaded on nanoparticles or in its free form. Analogous to free hypericin, cell killing induced by nanoparticle-loaded hypericin seems to be a function of cellular hypericin concentration and illumination times (Figure 7B).

## 3. Discussion

In this study, we investigated the phototoxic effects of free hypericin and nanoparticle-bound hypericin on the T cell leukemia cell line Jurkat and the colon carcinoma cell line HT-29 in dependence on concentration and illumination time. As shown here, the cellular uptake of hypericin was dose and time dependent. Hypericin mainly accumulated in the cytoplasm, probably in the endoplasmic reticulum and in the Golgi apparatus, as well as in lysosomes [19,20,28] (Figure 2). As long as treatment was performed in the absence of light, no acute toxic effects were observed. Only a weak influence of hypericin on cell proliferation was detected under these conditions (Figure 4 and Figure 5), which is in concordance with reported effects on cells even in the absence of light [5,15]. Due to its delocalized π-electron system, hypericin possesses photoactive properties and is able to produce ROS by energy transition [6,7,8]. If there is an increase of ROS, glutathione is oxidized to glutathion disulfide in order to protect the cell [33]. We showed that illuminated hypericin within tumor cells leads to ROS production and oxidative stress as proven by DCFH-DA staining (Figure 3). In parallel, glutathion was oxidized to glutathion disulfide, as verified by monobromobimane staining (Figure 3). In cases of low hypericin concentrations and short illumination times, the cellular antioxidative system is able to deal with oxidative stress. However, with increasing amounts of hypericin and illumination time, ROS overwhelms the cellular antioxidative systems and the amount of oxidizable glutathione is not sufficient any more. In this case, cellular organelles are damaged and subsequently, cell death is induced, dependent on hypericin concentration and illumination time. This is in agreement with earlier publications showing a shift from apoptosis to necrosis at increasing hypericin concentrations [4,18].

For tumor treatment with hypericin as a photosensitizer, it is crucially important that there is no remaining proliferation of tumor cells after the treatment, as it was shown for low concentrations and/or low light doses (Figure 4 and Figure 5). Moreover, it is important that tumor cells inactivated by PDT are recognized by the immune system. It has recently been reported that photodynamic therapy can induce immunogenic cell death and foster an anti-tumor response from the immune system [26,27]. For PDT, a multitude of immunogenic factors has been identified previously, which are either exposed on the plasma membrane (calreticulin, ecto-HSP70), actively secreted during early phases of cell death (ATP, HSP70), or passively released during final necrosis (HMGB1, HSP70). In accordance, we showed that illumination increased the number of dead cells after hypericin-treatment, resulting in an enhanced uptake of affected cells and dead material by macrophages (Figure 6). Further experiments are necessary to investigate the effect on various primary immune cells, upregulation of costimulatory molecules, and long-term outcome under in vivo conditions.

To further increase the efficacy and selectivity of cancer treatment and reduce side effects at the same time, nanotechnology comes more and more into focus. We previously showed that MDT can lead to a higher and especially selective drug accumulation in the tumor compared to conventional chemotherapy, whereas healthy tissues and the immune system are preserved from toxic side effects [21,22]. By combining MDT with PDT, a very selective therapy using a double-targeting strategy might be achieved. Hence, to generate toxicity of the drug, magnetic guidance and light irradiation must overlap. We previously described a method to load dextran-coated SPION with hypericin (SPION^Hyp^) for combined application in MDT and PDT [25]. As an outlook for future applications of SPION^Hyp^ in MDT we compared the cellular uptake and treatment efficacy of free hypericin, SPION^Hyp^, and SPIONs + free hypericin (Figure 7A). Compared to free hypericin, SPION^Hyp^ displayed a delayed cellular uptake due to diffusion processes in contrast to endocytosis. However, a sufficient illumination time of SPION^Hyp^-containing cells resulted in a complete stop of cell proliferation and induction of cell death. In summary, similar to the free substance, the efficacy of nanoparticle-loaded hypericin was dependent on drug accumulation and illumination time, showing a first promising prospect of a therapy combining MDT and PDT. Certainly, the in vitro findings should be verified in animal cancer models to address complex in vivo phenomena such as biodistribution, magnetic accumulation, and systemic effects of SPION^Hyp^. Based on the presented in vitro data, we conclude for the present that a double targeting strategy, namely magnetic accumulation and laser induced photoactivation might improve treatment effectivity and specificity and might reduce toxic side effects in future clinical applications.

## 4. Materials and Methods

### 4.1. Hypericin and Hypericin-Lloaded Superparamagnetic Iron Oxide Nanoparticles (SPION^Hyp^)

Hypericin (4,4′,5,5′,7,7′-hexahydroxy-2,2′-dimethylnaphthodianthrone) purchased by Alfa Aesar (Thermo Fisher Scientific, Karlsruhe, Germany) was dissolved in methanol (Carl Roth, Karlsruhe, Germany) resulting in a stock concentration of 75 μg/mL, which was stored at 4 °C in the dark until use. Hypericin bearing superparamagnetic iron oxide nanoparticles (SPIONs) were synthesized as described previously by Unterweger et al. [25] and extensively physicochemically characterized. In brief, SPIONs had a hydrodynamic diameter of 66.44 ± 2.2 nm and a ζ potential of −35.9 ± 1.1 mV. After loading with hypericin (SPION^Hyp^), their hydrodynamic size was 84.5 ± 0.3 nm and their ζ potential −40.7 ± 1.5 mV. For experiments concerning SPIONs, a hypericin concentration of 0.4 μM was chosen. The corresponding iron concentration of SPION^Hyp^ was 30 μg/mL.

### 4.2. Cells and Culture Conditions

The adherent colon carcinoma cell line HT-29 (ATCC/LGC GmbH, Wesel, Germany) and the non-adherent T cell leukemia cell line Jurkat (ACC 282, DSMZ, Braunschweig, Germany) were used. HT-29 cells were cultured in McCoy’s 5A medium (Gibco^®^, Life Technologies GmbH, Darmstadt, Germany) supplemented with 10% fetal calf serum (FCS), Jurkat cells were cultivated in RPMI 1640 medium supplemented with 10% FCS, and 1% glutamine (all from Thermo Fisher Scientific, Waltham, MA, USA). All cells were kept under standard cell culture conditions in a humidified incubator (INCOmed, Schwabach, Memmert, Germany) at 37 °C and 5% CO_2_. The cells were passaged twice a week and regularly checked for mycoplasma contamination using a PCR kit Venor^®^GeM (Minerva Biolabs GmbH, Berlin, Germany). To count cells and determine viability, MUSE^®^Cell Analyzer (Merck-Millipore, Billerica, MA, USA) was used.

### 4.3. Hypericin Treatment of Cells

HT-29 cells were adjusted to a density of 5 × 10^5^ cells/mL and 1 mL cell suspension was seeded into 24-well plates (TPP Techno Plastic Products AG, Trasadingen, Schweiz) and incubated for adherence overnight. Jurkat cells were adjusted to a density of 2.5 × 10^5^ cells/mL and 1 mL was seeded into 48-well plates (Greiner bio-one, Frickenhausen, Germany). Then, cells were pre-incubated with free hypericin for 16 h in the dark (plates were wrapped with aluminum foil) to enable uptake by the cells. Methanol-treated cells and phosphate buffered saline (PBS)-treated cells served as controls. Then, cells were illuminated with a slimlite light-emitting diode (LED) white light source (40 W/m^2^, Kaiser, Buchen, Germany) for various time intervals to start the phototoxic reaction of hypericin.

### 4.4. Fluorescence Microscopy

Glass slides (Menzel-Gläser, Carl Roth, Karlsruhe, Germany) were coated overnight with 200 μL/cm^2^ 1.5% gelatin (Sigma Aldrich, Steinheim, Germany) in PBS in 24-well plates. The next day, unbound gelatin was removed and slides were washed with PBS. Next, 1 × 10^5^ HT-29 cells were seeded onto the slides, and incubated for adherence. After treatment of cells with hypericin overnight, cells were washed with PBS and fixed with 500 μL 3% PBS-FA (formaldehyde 37%, Carl Roth, Karlsruhe) in each well for 15 min. Then, cells were washed with PBS and plasma membrane was permeabilized with 500 μL Triton X-100 in PBS for 5 min. Then, 5% horse serum (GE Healthcare, Dornstadt, Germany) was added for 30 min to block the nonspecific binding sites. After washing with PBS, cells were stained with 100 μL of Phalloidin-FITC in PBS (Life Technologies Carlsbad, CA, USA; 1:125) by incubating for 1 h in the dark and washing with PBS afterwards. After that, cells were stained with 250 μL of 80 μg/mL Hoechst 33342 (Life Technologies, Carlsbad, CA, USA) for 15 min. Subsequently, cells were washed with PBS. Hoechst (excitation 405 nm; emission 430 nm) and hypericin (excitation 488 nm; emission 595 nm) were analyzed in fluorescence microscopy (Axio Observer Z.1 microscope; Carl Zeiss AG, Oberkochen, Germany). Hypericin fluorescence was analyzed by ZEN pro 2012 software (Carl Zeiss AG, Oberkochen, Germany) and pictures were edited in Photoshop software (version CS6, Adobe, San Jose, CA, USA).

### 4.5. Analysis of Viability and Drug Penetration into Cells by Flow Cytometry

Fifty microliter (50 μL) aliquots of cells were stained with 250 μL staining mix consisting of 1 mL Ringer’s Solution (Delta Select, Rimbach, Germany), 0.33 μL monobromobimane (MBB, Sigma-Aldrich, Hamburg, Germany, stock conc. 100 mM), and 0.4 μL hexamethylindodicarbocyanine iodide dye (DiIC_1_(5)) (supplied by Thermo Fisher Scientific, Waltham, MA, USA) and incubated for 30 min at 4 °C. Cells were analyzed in a Gallios flow cytometer (Beckman Coulter, Fullerton, CA, USA). Hypericin was excited at 488 nm, and recorded by FL 3 sensor (620/30 nm BP). MBB fluorescence was excited at 405 nm and recorded on the FL9 sensor (430/40 nm BP) and DiIC_1_(5) was excited at 638 nm and recorded on an FL6 sensor (675/20 nm BP). In order to eliminate fluorescence bleed-through, electronic compensation was used. Data were analyzed with KaluzaTM software Version 1.2 (Beckman Coulter, Fullerton, CA, USA) and further processed in Microsoft Excel (version 2010, Microsoft, Redmont, Washington, DC, USA). 

### 4.6. Cell Proliferation after Treatment with Hypericin

HT-29 cells were adjusted to a density of 5 × 10^5^ cells /mL and 1 mL of cell suspension was seeded per well in 24-well plates (TPP Techno Plastic Products AG, Trasadingen, Schweiz). After attachment of the cells, pre-incubation with hypericin, and light illumination, the cell proliferation was monitored for 72 h using the IncuCyte^®^ device (Essen BioScience, Ann Arbor, MI, USA) and cell confluence was calculated from the microscopy images.

### 4.7. Analysis of ROS Generation Using DCFH-DA

Jurkat cells were stained at a density of 1 × 10^6^ cells/mL in 10 mL PBS with 40 μM DCFH-DA (Dichlorodihydrofluorescein diacetate, Sigma-Aldrich, Hamburg, Germany) for 30 min at 37 °C. After washing with PBS, 1.5 mL cells were pipetted into 48-well plates (Cellstar Greiner Bio-One, Frickenhausen, Germany) and treated with hypericin. For measurement of ROS, 100 μL of cells were transferred into white Lumitrae 96-well plates (Greiner bio-one, Frickenhausen, Germany) and illuminated. Immediately after illumination, generation of ROS was monitored in Filtermax F5 Photometer (excitation 488 nm; emission 535 nm, Molecular Devices, CA, USA). For microscopic analysis of ROS generation, HT-29 cells were stained with 150 μL/well staining dilution consisting of 16.2 mM Hoechst 33342 and 40 μM DCFH in 10 mL Ringer’s solution for 30 min at 37 °C. After illumination, cells were washed with PBS and glass slides were fixed with mounting medium (Dako Fluorescent Mounting Medium Carpenteria, CA, USA). Fluorescence microscopy was performed as described previously.

### 4.8. Phagocytosis of Hypericin-Treated Cells by Macrophages

THP-1 cells (ATCC) were cultured in RPMI 1640 medium (Biochrom, Berlin, Germany) supplemented with 2 mmol/L glutamine, 100 U/mL penicillin, 100 μg/mL streptomycin, and 10% fetal calf serum. For staining of the plasma membrane, THP-1 cells were adjusted to a density of 1 × 10^5^ cells/mL in 2 mL FCS-free Medium and incubated with 5 μL DiD (Thermo Fisher Scientific, Waltham, MA, USA) per mL cell suspension. After 20 min incubation at 37 °C, the reaction was stopped with THP-1 medium containing FCS and cells were washed twice. To differentiate cells into macrophages, 10^5^ cells/mL were seeded in 24-well plates in the presence of 0.02 μg/mL phorbol 12-myristate 13-acetate (PMA) (Sigma-Aldrich, Hamburg, Germany) for 72 h. After 72 h, PMA-containing medium was removed and replaced by PMA-free medium for 24 h.

Jurkat cells were adjusted to a density of 2.5 × 10^5^/mL in PBS and stained with 10 μM CFSE for 15 min at 37 °C. Staining reaction was stopped with cell culture medium and cells were washed twice. To allow cells to pump excess dye, cells were incubated for 6 h in cell culture medium, followed by washing them twice with PBS before seeding them into 40 mL FCS-free medium. Then cells were loaded with hypericin. Immediately after illumination, 500 μL Jurkat cells (1.25 × 10^5^) were added as “prey” to differentiated macrophages. After washing with PBS, THP-1 cells were either analyzed in fluorescence microscopy or detached from the wells for flow cytometry using trypsin.

### 4.9. Comparison of Jurkat Cells Treated with Free Hypericin and SPION^Hyp^

Cells were seeded in a 48-well-plate to a density of 2 × 10^5^ cells/mL in culture medium. Cells were treated with 50 μL of 0.4 μM hypericin, SPION^Hyp^, or SPION + free hypericin. Unloaded SPION, ethanol, and water served as controls. After 2 h incubation in the dark at 37 °C, cells were illuminated as described previously and measurements were done at *t* = 16 h after illumination. For flow cytometry analysis, cells were stained with 250 μL of staining solution as described above.

## 5. Conclusions

Hypericin in free and nanoparticle bound form (SPION^Hyp^) for magnetic drug targeting was dose and time dependently taken up by cells. In the absence of light, we observed no acute toxicity. Phototoxic effects were caused by ROS, overwhelming the cellular antioxidative system and were dependent on hypericin concentration and light illumination time. Comparing efficacy of SPION^Hyp^ and free hypericin, the uptake of SPION^Hyp^ by cells was slowlier and the phototoxic effects therefore accordingly weaker, so that for SPION^Hyp^ hypericin concentration and/or illumination times must be adapted. For future tumor therapy, a double targeting strategy, namely magnetic accumulation of SPION^Hyp^ in the tumor region and laser induced photoactivation might improve treatment efficacy and specificity and might reduce systemic toxic side effects.

## Abbreviations

SEON	Section of Experimental Oncology and Nanomedicine
SPIONs	Superparamagnetic iron oxide nanoparticles
PDT	Photodynamic therapy
MDT	Magnetic drug targeting
ROS	Reactive oxygen species
ER	Endoplasmic reticulum
DCFH-DA	2′,7′-Dichlorodihydrofluorescein diacetate
MBB	Monobromobimane
GSH	Glutathione
GSSG	Glutathione disulfide
MeOH	Methanol
DiIC_1_(5)	hexamethylindodicarbocyanine iodide dye
FSc	Forward scatter
SSc	Side scatter
DiD	C_67_H_103_ClN_2_O_3_S
CFSE	Carboxyfluorescein succinimidyl ester
SPION^Hyp^	Hypericin loaded superparamagnetic iron oxide nanoparticles
Hyp	Hypericin
DAMPs	Damage-associated molecular patterns
FCS	Fetal calf serum
PCR	Polymerase chain reaction
PBS	Phosphate buffered saline
LED	Light-emitting diode
FA	Formaldehyde
Fitc	Fluorescein isothiocyanate
FL	Fluorescence
PMA	Phorbol 12-myristate 13-acetate
MFI	Mean fluorescence index
ctrl	Control
